# Quantitative bone SPECT/CT reconstruction utilizing anatomical information

**DOI:** 10.1186/s40658-020-00348-1

**Published:** 2021-01-06

**Authors:** Tuija S. Kangasmaa, Chris Constable, Antti O. Sohlberg

**Affiliations:** 1 Department of Clinical Physiology and Nuclear Medicine, Vaasa Central Hospital, Hietalahdenkatu 2-4, 65130 Vaasa, Finland; 2 HERMES Medical Solutions, Strandbergsgatan 16, 11251 Stockholm, Sweden; 3 Laboratory of Clinical Physiology and Nuclear Medicine, Päijät-Häme Central Hospital, Keskussairaalankatu 7, 15850 Lahti, Finland

**Keywords:** Bone SPECT/CT, Bayesian reconstruction, Anatomical prior, Quantitation

## Abstract

**Background:**

Bone SPECT/CT has been shown to offer superior sensitivity and specificity compared to conventional whole-body planar scanning. Furthermore, bone SPECT/CT allows quantitative imaging, which is challenging with planar methods. In order to gain better quantitative accuracy, Bayesian reconstruction algorithms, including both image derived and anatomically guided priors, have been utilized in reconstruction in PET/CT scanning, but they have not been widely used in SPECT/CT studies. Therefore, the aim of this work was to evaluate the performance of CT-guided reconstruction in quantitative bone SPECT.

**Methods:**

Three Bayesian reconstruction methods were evaluated against the conventional ordered subsets expectation maximization (OSEM) reconstruction method. One of the studied Bayesian methods was the relative difference prior (RDP), which has recently gained popularity in PET reconstruction. The other two methods, anatomically guided smoothing prior (AMAP-S) and anatomically guided relative difference prior (AMAP-R), utilized anatomical information from the CT scan. The reconstruction methods were evaluated in terms of quantitative accuracy with artificial lesions inserted in clinical patient studies and with 20 real clinical patients. Maximum and mean standardized uptake values (SUVs) of the lesions were defined.

**Results:**

The analyses showed that all studied Bayesian methods performed better than OSEM and the anatomical priors also outperformed RDP. The average relative error in mean SUV for the artificial lesion study for OSEM, RDP, AMAP-S, and AMAP-R was − 53%, − 35%, − 15%, and − 10%, when the CT study had matching lesions. In the patient study, the RDP method gave 16 ± 9% higher maximum SUV values than OSEM, while AMAP-S and AMAP-R offered increases of 36 ± 8% and 36 ± 9%, respectively. Mean SUV increased for RDP, AMAP-S, and AMAP-R by 18 ± 9%, 26 ± 5%, and 33 ± 5% when compared to OSEM.

**Conclusions:**

The Bayesian methods with anatomical prior, especially the relative difference prior-based method (AMAP-R), outperformed OSEM and reconstruction without anatomical prior in terms of quantitative accuracy.

## Background

Bone scanning is an invaluable tool in screening and follow-up of bone metastases from primary breast and prostate cancers. Conventionally, bone scanning has been accomplished by whole-body planar scanning, but the sensitivity and specificity of SPECT and especially SPECT/CT have been reported to be superior to planar imaging [1]. In addition to improved lesion detection, bone SPECT/CT allows quantitative and semi-quantitative imaging: for example, the images can be reported in standardized uptake values (SUVs) analogous to routine PET imaging [2]. Here, SUV is defined as:

1 $$ \mathrm{SUV}=\frac{C_{\mathrm{ROI}}}{A_{Inj}/w} $$

where *C*_ROI_ is the activity concentration [kBq/ml] within a region of interest (ROI), *A*_*Inj*_ is the decay-corrected injected activity [kBq], and *w* is the weight of the patient [g].

Quantitative SPECT has been commercially available for several years now, but it has not yet been widely applied. Only a couple of approaches based on quantitative SPECT, mainly in the field of dosimetry after radionuclide therapy, have been clinically accepted [3]. The lack of acceptance is partly due to poorer spatial resolution of SPECT/CT when compared to PET/CT, which results in inferior quantitative performance. Quantitative bone SPECT/CT, however, holds promise in aiding patient follow-up and interpatient comparisons. It might also help in the interpretation of bone SPECT studies, e.g., by allowing global scaling based on SUV, thus removing scaling difficulties due to hot bladder, or it might provide easier detection of the super-scan phenomenon [4], because there is no need to relate bone uptake to kidney uptake.

Computed tomography is currently used only for attenuation compensation and anatomical localization in bone SPECT/CT. Anatomical information derived from CT could also be utilized to guide the SPECT image reconstruction algorithm and therefore help reduce the noise and image blurring. Anatomically guided reconstruction algorithms have been tested in brain PET/MRI [5, 6], but they have not found their way to clinical SPECT/CT image reconstruction yet. Therefore, the aim of this work was to evaluate the performance of CT-guided reconstruction in quantitative bone SPECT.

Emission image reconstruction using the maximum likelihood expectation maximization (ML-EM) algorithm usually does not make assumptions about how the image should look beforehand. However, it is possible to incorporate some a priori knowledge into the reconstruction, such as the expected image smoothness or anatomical knowledge from a registered CT or MRI. For this purpose, the maximum likelihood expectation maximization algorithms, which obtain the reconstructed image by directly fitting to measured projection data, have to be replaced by Bayesian methods, where the image is reconstructed by maximizing the posteriori distribution consisting of the likelihood data fitting term and prior term. Several Bayesian methods utilizing anatomical information have been presented [7]. Many of them require image segmentation into different tissue classes or knowledge of edge positions, which can be difficult to accomplish in clinical practice. We selected two easily tunable anatomical Bayesian reconstruction methods and compared them against the conventional ordered subsets expectation maximization (OSEM) algorithm and the relative difference prior (RDP), which is a Bayesian reconstruction method not utilizing anatomical prior information. RDP was included due to its current success in PET reconstruction [8].

## Methods

### Patient studies with artificial lesions

To simulate lesion-present clinical studies in which the presence, location, and tracer uptake of lesions were known, 15 mm and 20 mm spherical lesions were added to disease-free whole-body bone SPECT/CT studies. Two (1 female, 76 kg, and 1 male, 81 kg) three-bed Tc99m-HDP bone SPECT/CT studies which had been reported as normal were selected from Lahti Central Hospital’s database. The studies were acquired on a Siemens Symbia T using 256 × 256 matrix size, 2.4 mm pixel size, 64 projection angles over 360° rotation, 20 s per acquisition angle, and a body contour orbit. The CT scan was performed in shallow free-breathing with 130 kV and 25 ref mAs. CT images were reconstructed into 512 × 512 matrix, with 0.98 mm pixel size and 3.0 mm slice thickness. The alignment between SPECT and CT was carefully validated according to clinical practice. Spherical lesions with SUV of 15 were then mathematically generated, projected using the projector presented in [9], which modeled attenuation and collimator/detector effects, and finally added to the original projection data. Knowing the lesions’ sizes were relatively small and well separated in the body, the number of lesion scatter counts was assumed to be low compared to scatter coming from other parts of the skeleton and modeling of additional scattered photons from the lesions was omitted. The same assumption is often used when simulating lesions in PET studies.

A total of five lesions—one each in the skull, sternum, ribs, spine, and pelvis—were added. The skull, sternum, and rib lesions were 15 mm diameter and the spine and pelvis lesions 20 mm diameter. The lesions were placed in bone areas where the CT image had uniform appearance representing cases where the bone lesion is only seen in the SPECT images. We also generated CT images, which had lesions with the same size, shape, and location as the SPECT lesions representing cases where the bone lesion is seen in both SPECT and CT images. In addition, we shifted both the lesion-absent and lesion-present CT images 5 mm in anterior-posterior direction to study the effects of SPECT-CT misalignment. The 5-mm shift was based on work published by Kuwert [3] which states that in the neck and in organs affected by respiratory motion, the average misalignment is usually less than 5 mm.

The artificial lesions were analyzed by placing spherical volumes of interest (VOIs) of the known diameter and location on the reconstructed images. SUV_max_ and SUV_mean_ for each lesion on both datasets were calculated and averaged over the two phantoms.

### Clinical patient studies

Twenty clinical three-bed Tc99m-HDP bone SPECT/CT studies performed on patients with breast and prostate cancer diagnoses (7 female, 13 male, mean age 75 years, mean weight 80 kg, mean injected activity 705 MBq) were randomly drawn from Lahti Central Hospital’s database. All the studies were acquired with Siemens Symbia T using the same acquisition parameters described earlier. Patient weights and information about injected activities and injection times were available, and thus, the studies could be reconstructed in SUV units according to Lahti Central Hospital’s clinical practice.

Spherical VOIs of 10 mm diameter were placed on center of focal hotspots (SUV_mean_ ≥ 15) in the areas of skull, sternum, ribs, spine, and pelvis. Up to five VOIs were placed on each area on every patient if lesions were visible. The VOI size was chosen so that the VOI included only bone tissue also in the narrow skull and rib uptake areas and did not expand outside bone seen in CT. No knowledge of patient history or laboratory results (e.g., elevated PSA or alkaline phosphates value, TNM classification, Gleason score, or location of possible bone pain) was available. Therefore, the hotspot VOIs correspond to both benign and malignant uptake areas. VOIs of the same size were also placed on normal bone on the skull, sternum, ribs, spine, and pelvis. Up to five VOIs were placed on each area on every patient if there was sufficient normal bone available. The normal uptake area was also selected based only on SUV. SUV_max_ and SUV_mean_ inside the VOI were extracted.

### Image reconstruction

All images (patient studies with artificial lesions and clinical patient studies) were reconstructed using HybridRecon v3.2 (HERMES Medical Solutions, Stockholm, Sweden) with algorithms based on the GPU-accelerated OSEM presented in [10]. OSEM is defined as:

2 $$ {f}_j^{\mathrm{new}}=\frac{f_j^{\mathrm{old}}}{\sum_{i\in {S}_n}{a}_{ij}}{\sum}_{i\in {S}_n}{a}_{ij}\frac{p_i}{\sum_k{a}_{ik}{f}_k^{\mathrm{old}}} $$

where *j* (and *k*) is the reconstruction voxel index, *i* the projection pixel index, *f*^new^ the new image estimate, *f*^old^ the old image estimate, *S*_*n*_ the subset *n*, *p* the projections, and *a*_*ij*_ the probability that photon emitted from voxel *j* (or *k*) is detected in projection pixel *i* [11]. *a*_*ij*_ is an element of the projector operator *A*, which in our case included CT-based attenuation model, distance-dependent 2D Gaussian collimator-detector model, and Monte Carlo-based scatter model [9]. OSEM images were post-filtered with a 3D Gaussian filter to suppress noise.

Bayesian reconstruction methods were based on the one step late (OSL) algorithm [12]:

3 $$ {f}_j^{new}=\frac{f_j^{old}}{\sum_{i\notin {S}_n}{a}_{ij}+\beta \frac{\partial U\left({f}^{old}\right)}{\partial {f}^{old}}}\sum \limits_{i\notin {S}_n}{a}_{ij}\frac{Pi}{\sum \limits_k{a}_{ik}{f}_k^{old}} $$

where *β* is the Bayesian weight and $\frac{\partial U\left({f}^{old}\right)}{\partial {f}^{old}}$ the derivative of the energy function *U*. Three energy functions were used. The first one was the RDP, whose energy function is defined as:

4 $$ U(f)=\sum \limits_j\sum \limits_{k\in {N}_j}\frac{{\left({f}_j-{f}_k\right)}^2}{\left({f}_j+{f}_k\right)+\gamma \left|{f}_j-{f}_k\right|} $$

where *N*_*j*_ is the neighborhood of voxel *j* and constant *γ* controls the degree of edge preservation [13]. Even though the plain RDP does not utilize anatomical information from a CT, it can still improve the resolution of the reconstructed images by preserving image edges. The second energy function was the anatomically guided smoothing prior (AMAP-S):

5 $$ U(f)=\sum \limits_j\sum \limits_{k\in B}{\left({f}_j-{f}_k\right)}^2 $$

where instead of using all voxels in the neighborhood *N*_*j*_ only *B* most similar voxels in neighborhood are used [14]. The most similar voxels are found by comparing the absolute differences in CT Hounsfield values. The third energy function was equal to RDP, but instead of using all voxels in the neighborhood *N*_*j*_, anatomical information from the CT was used to pick the *B* most similar neighbors. This algorithm is called AMAP-R. A 3 × 3 × 3 voxel neighborhood was used with all energy functions.

**Fig. 1:**
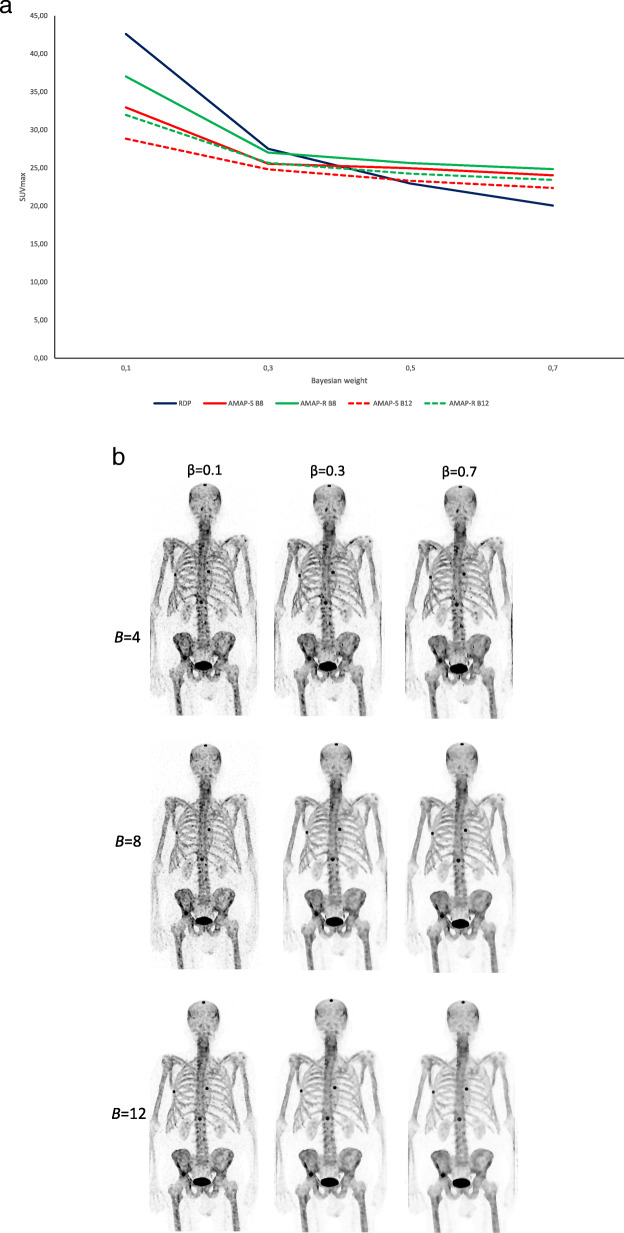
**a**. Maximum SUV of the pelvic lesion as a function of Bayesian weight (*β*) for RDP (blue line), AMAP-S (red line) and AMAP-R (green line). AMAP-S and AMAP-R results are presented with two numbers of most similar neighbours B=8 (solid line) and B=12 (dashed line). **b**. Effect of Bayesian weight (*β*) and number of most similar neighbors (*B*) on image quality for AMAP-S. Effects for RDP and AMAP-R are similar. Anterior maximum intensity projection is shown

The number of subsets, iterations, Bayesian weight *β*, constant *γ*, and the number of most similar neighbors *B* were determined by reconstructing bone SPECT/CT studies using a wide range of the aforementioned parameters. A compromise in terms of image quality, lesion SUV, and noise suppression had to be made by comparing the Bayesian reconstructed images to clinical OSEM images (5 iterations, 16 subsets, and 9 mm full width at half maximum (FWHM) 3D Gaussian post-filter), because some of the parameters are counteracting. In general, increase in Bayesian weight or number of most similar neighbors leads to decreased noise, lower resolution, and more blocky appearance of the images, whereas larger constant *γ* produce sharper, but noisier images. The effect of *β* and *B* on maximum SUV is presented in Fig. 1a and on image quality and in Fig. 1b. Chosen reconstruction parameters are presented in Table 1.

**Table 1:** Reconstruction parameters

Parameter	OSEM	RDP	AMAP-S	AMAP-R
Iterations	5	15	15	15
Subsets	16	16	16	16
FWHM [mm]	9	–	–	–
*β*	–	0.3	0.3	0.3
*γ*	–	3.0	–	3.0
*B*	–	–	8	8

## Results

### Patient studies with artificial lesions

**Fig. 2:**
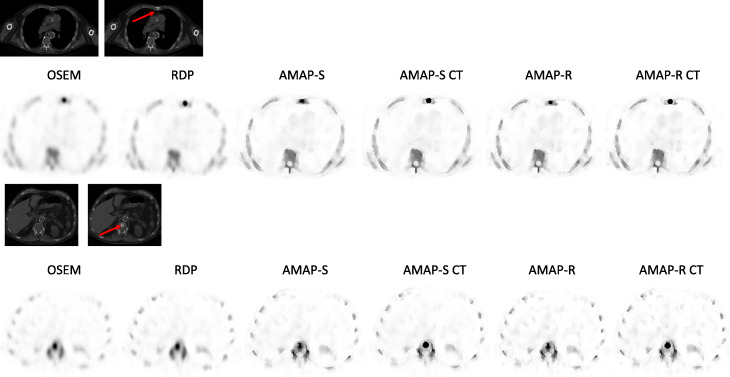
Example reconstructed SPECT and CT (with and without lesions) transverse slices at the level of sternum and spine lesions of the male patient study with artificial lesions. Red arrows show the lesion locations. VOI used to analyze the reconstructed SPECT studies had the same shape and position as the lesion seen on the CT image. AMAP-S and AMAP-R refer to reconstructions where lesion is absent in the CT and AMAP-S CT and AMAP-R CT to lesion-present cases. The color scale is set to SUV 15 for all SPECT images

**Fig. 3:**
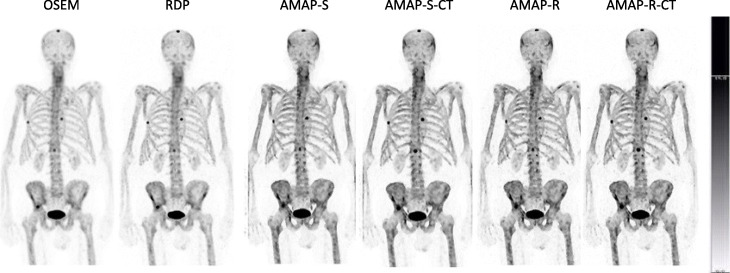
Anterior maximum intensity projections of the male patient study with artificial lesions. AMAP-S and AMAP-R refer to reconstructions where lesion is absent in the CT and AMAP-S CT and AMAP-R CT to lesion-present cases. The color scale is set to SUV 15 for all images

Table 2 presents SUV_max_ and SUV_mean_ for the artificial lesions for the four reconstruction methods. The three Bayesian methods provide SUV_mean_ values, which are closer to the true SUV value (SUV = 15) than OSEM for all lesions. The improvement in SUV_mean_ accuracy is dependent on the lesion location, and interestingly, RDP partly provides more accurate SUV_mean_ estimates than AMAP-S in cases where the lesion is not present in the CT. AMAP-R offers the largest improvement independent of the lesion location. Reconstructions with lesion-present CTs perform considerably better than reconstructions with lesion-free CTs. Lesion-present CTs also render AMAP-S and AMAP-R lesions into the correct shape and size as can be seen from Figs. 2 and 3. SUV_max_ values overshoot the true SUV value with all Bayesian reconstruction methods. AMAP-S and AMAP-R reconstructions with lesion-present CTs (AMAP-S CT and AMAP-R CT) partly reduce this overshoot, but cannot completely mitigate it. Average relative error (= 100 × (SUV_mean_ − 15)/15) in SUV_mean_ for OSEM, RDP, AMAP-S, AMAP-S CT, AMAP-R, and AMAP-R CT is − 53%, − 35%, − 40%, − 15%, − 30%, and − 10%.

**Table 2:** SUV_max_ and SUV_mean_ (mean ± standard deviation) for the 4 different reconstruction algorithms. AMAP-S and AMAP-R refer to reconstructions where lesion is absent in the CT and AMAP-S CT and AMAP-R CT to lesion-present cases

Method	SUV	Area				
		Skull	Sternum	Ribs	Spine	Pelvis
**OSEM**	Max	13.3 ± 1.2	11.4 ± 1.3	13.0 ± 0.2	9.2 ± 0.3	18.6 ± 3.6
	Mean	7.6 ± 0.1	7.2 ± 0.1	6.8 ± 0.1	4.9 ± 0.7	8.7 ± 1.1
**RDP**	Max	21.2 ± 4.1	20.8 ± 2.6	24.8 ± 2.1	10.2 ± 0.1	23.1 ± 6.3
	Mean	11.3 ± 1.3	11.5 ± 0.7	10.5 ± 0.8	4.9 ± 0.3	10.2 ± 2.1
**AMAP-S**	Max	22.9 ± 1.6	32.2 ± 6.7	26.3 ± 1.0	10.9 ± 7.8	22.2 ± 4.0
	Mean	9.4 ± 1.3	11.4 ± 1.1	8.9 ± 0.2	5.4 ± 0.3	10.0 ± 1.1
**AMAP-S CT**	Max	31.2 ± 3.4	18.4 ± 5.1	17.4 ± 3.1	11.9 ± 2.4	17.5 ± 2.6
	Mean	16.2 ± 0.1	12.3 ± 1.9	11.3 ± 1.4	11.0 ± 2.8	12.8 ± 0.3
**AMAP-R**	Max	34.3 ± 5.3	37.2 ± 2.9	45.0 ± 7.3	16.0 ± 8.7	29.7 ± 2.0
	Mean	12.1 ± 2.8	12.2 ± 0.3	11.2 ± 0.3	6.0 ± 0.2	11.0 ± 1.2
**AMAP-R CT**	Max	35.9 ± 1.5	16.4 ± 8.2	21.9 ± 2.5	13.5 ± 1.3	19.8 ± 3.2
	Mean	17.7 ± 0.9	12.7 ± 1.6	12.4 ± 1.7	10.9 ± 1.4	14.1 ± 0.2

**Fig. 4:**
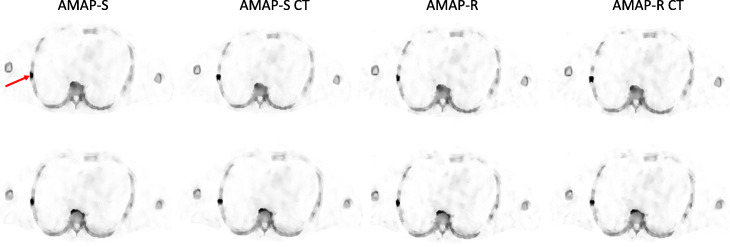
Example reconstructed SPECT transverse slice for AMAP-S and AMAP-R with and without the 5-mm SPECT/CT mismatch at the level of rib lesion of the male patient study with artificial lesions. Red arrow shows the lesion location. AMAP-S and AMAP-R refer to reconstructions, where lesion is absent in the CT, AMAP-S CT, and AMAP-R CT to lesion-present cases. Top row shows aligned cases and bottom row reconstructions with the 5-mm SPECT/CT mismatch. The color scale is set to SUV 15 for all images

**Fig. 5:**
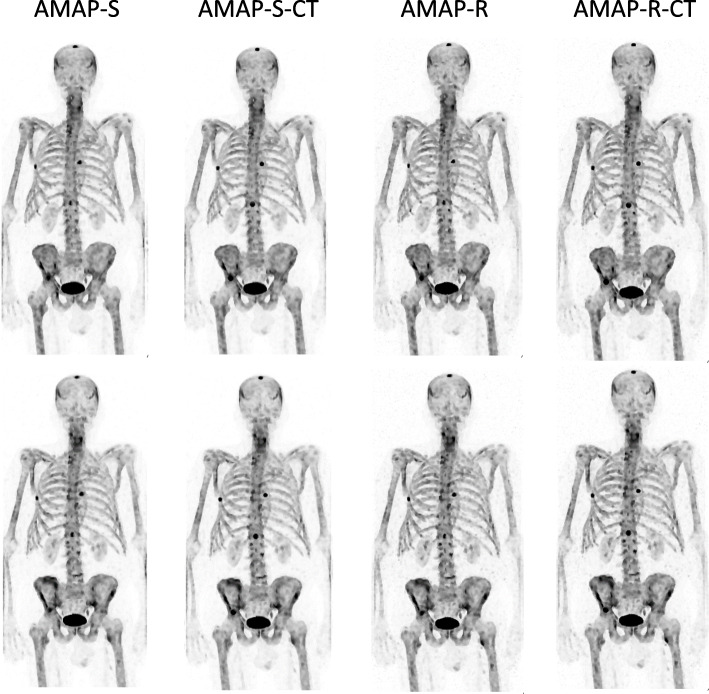
Anterior maximum intensity projections of the male patient study with artificial lesions with SPECT/CT mismatch. AMAP-S and AMAP-R refer to reconstructions where lesion is absent in the CT and AMAP-S CT and AMAP-R CT to lesion-present cases. Top row shows perfectly aligned cases and bottom row reconstructions with the 5-mm SPECT/CT mismatch. The color scale is set to SUV 15 for all images

The effect of SPECT/CT position mismatch is presented in Table 3. AMAP-S and AMAP-R reconstructions are more sensitive to SPECT/CT mismatch than OSEM or RDP. This is especially true for reconstructions with matching CT lesions. The difference generated by the 5-mm mismatch is however difficult to appreciate visually as shown in Figs. 4 and 5.

**Table 3:** Relative difference (100% × (SUV_mean_true_ − SUV_mean_5mm_)/SUV_mean_true_) in SUV_mean_ values for the 4 different reconstruction algorithms generated by 5 mm mismatch between SPECT and CT. AMAP-S and AMAP-R refer to reconstructions where lesion is absent in the CT and AMAP-S CT and AMAP-R CT to lesion-present cases

Method	Area				
	Skull (%)	Sternum (%)	Ribs (%)	Spine (%)	Pelvis (%)
**OSEM**	− 5.6	13.2	4.6	2.5	6.2
**RDP**	− 5.5	11.9	5.0	3.8	5.4
**AMAP-S**	5.3	35.7	6.8	− 14.0	10.3
**AMAP-S CT**	30.3	26.2	15.6	30.0	12.7
**AMAP-R**	7.1	28.9	3.4	− 14.5	7.6
**AMAP-R CT**	20.2	24.7	11.7	31.4	18.9

### Clinical patient studies

**Fig. 6:**
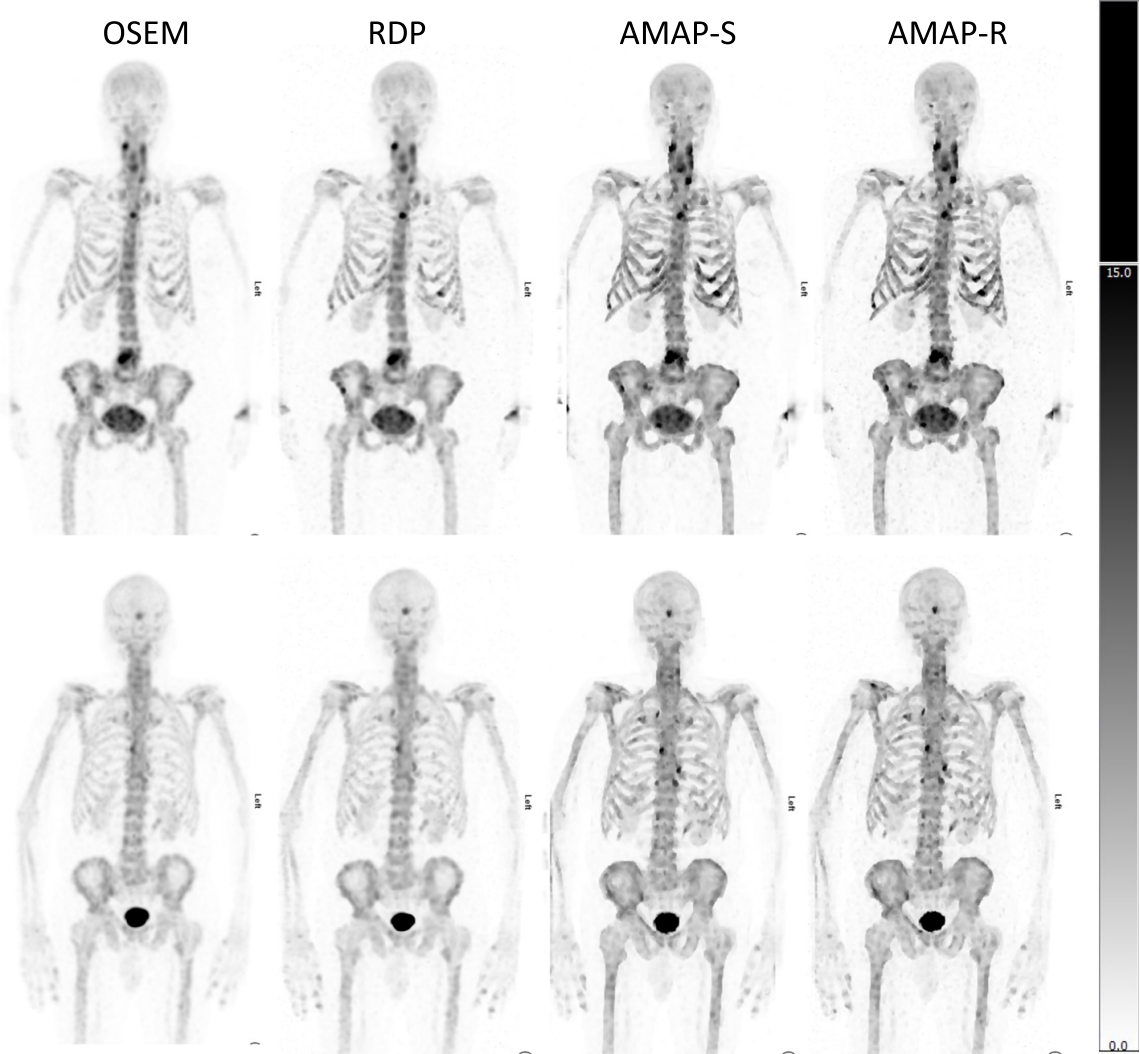
Anterior MIP of two clinical patient studies (first study in top row and second in the bottom). The color scale is set to SUV 15 for all images

**Fig. 7:**
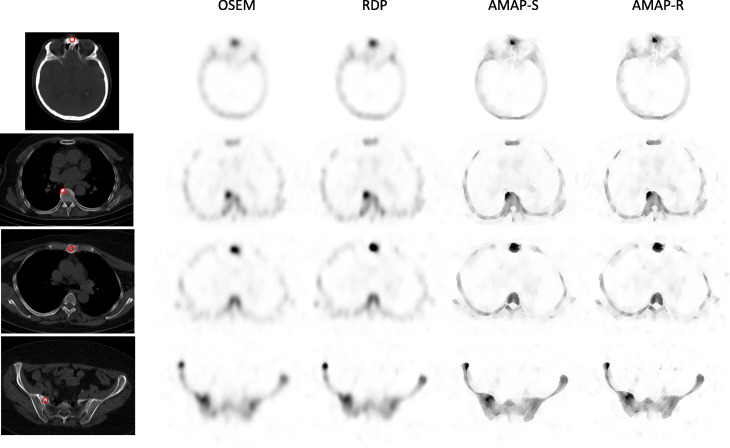
Example transversal slices of four lesions from clinical patient studies. The red circle presents the VOI used to analyze the studies. The color scale is set to SUV 15 for all images

The number of lesions and normal uptake samples for each area is listed in Table 4. Table 5 shows SUVs for the lesions, Table 6 for normal uptake areas, and Table 7 for SUVR, which was defined as the ratio of lesion and normal uptake SUVs. Tables 5 and 7 show that Bayesian methods offer higher SUVs than OSEM also with clinical patient data. For SUV_max_, the RDP method gives 16 ± 9% higher values than OSEM, while AMAP-S and AMAP-R offer increases of 36 ± 8% and 36 ± 9%, respectively. With regard to SUV_mean_ RDP, AMAP-S and AMAP-R offer increases of 18 ± 9%, 26 ± 5%, and 33 ± 5%, respectively. These differences to OSEM are statistically significant (Wilcoxon signed rank test), with all *p* values less than 0.001. Figures 6 and 7 show example images of clinical patient studies. Figure 6 shows example maximum intensity projections (MIP) of two clinical patients, while Fig. 7 shows example transversal slices of two lesion areas for the same two patients. AMAP-S and AMAP-R images clearly show more anatomical detail when compared to OSEM or RDP.

**Table 4:** Number of lesions (*n*_lesion_) and normal uptake samples (*n*_normal_) for the clinical patient studies

Area	***n***_**lesion**_	***n***_**normal**_
Skull	3	100
Sternum	5	95
Ribs	3	100
Spine	19	100
Pelvis	14	100

**Table 5:** Lesion SUV_max_ and SUV_mean_ values (mean ± standard deviation) for the clinical data

Method	SUV	Area				
		Skull	Sternum	Ribs	Spine	Pelvis
OSEM	Max	13.6 ± 2.0	15.5 ± 1.0	15.0 ± 1.5	20.6 ± 2.3	17.3 ± 2.6
	Mean	11.3 ± 1.5	12.7 ± 1.0	11.6 ± 0.9	16.9 ± 2.1	14.3 ± 2.3
RDP	Max	16.3 ± 2.4	19.9 ± 1.4	22.6 ± 2.9	22.6 ± 2.6	19.3 ± 3.3
	Mean	13.7 ± 1.6	16.3 ± 1.3	17.3 ± 1.5	19.0 ± 2.4	16.4 ± 2.8
AMAP-S	Max	23.1 ± 3.0	26.1 ± 1.3	32.5 ± 2.7	28.5 ± 3.3	25.8 ± 3.4
	Mean	17.6 ± 1.7	19.7 ± 1.8	22.2 ± 1.5	22.4 ± 2.2	20.4 ± 2.4
AMAP-R	Max	24.6 ± 2.9	27.0 ± 1.5	35.7 ± 5.6	30.9 ± 3.7	27.0 ± 4.2
	Mean	19.2 ± 1.7	20.3 ± 1.8	23.5 ± 2.1	23.9 ± 2.3	21.6 ± 2.7

**Table 6:** Normal uptake SUV_max_ and SUV_mean_ (mean ± standard deviation) for the clinical data

Method	SUV	Area				
		Skull	Sternum	Ribs	Spine	Pelvis
OSEM	Max	1.6 ± 0.2	3.8 ± 0.3	2.0 ± 0.1	4.8 ± 0.4	4.6 ± 0.3
	Mean	1.3 ± 0.2	3.0 ± 0.2	1.5 ± 0.1	3.7 ± 0.3	3.7 ± 0.2
RDP	Max	1.7 ± 0.2	4.1 ± 0.3	2.3 ± 0.1	4.8 ± 0.4	4.8 ± 0.4
	Mean	1.4 ± 0.2	3.1 ± 0.2	1.6 ± 0.1	3.8 ± 0.4	3.8 ± 0.2
AMAP-S	Max	2.3 ± 0.2	5.1 ± 0.3	3.2 ± 0.3	5.0 ± 0.2	4.9 ± 0.4
	Mean	1.7 ± 0.2	3.7 ± 0.3	2.2 ± 0.2	3.9 ± 0.2	3.9 ± 0.2
AMAP-R	Max	2.4 ± 0.2	5.1 ± 0.3	3.4 ± 0.4	4.9 ± 0.3	5.0 ± 0.5
	Mean	1.7 ± 0.2	3.7 ± 0.3	2.2 ± 0.2	3.8 ± 0.2	4.0 ± 0.2

**Table 7:** SUVR_max_ and SUVR_mean_ (SUVR = SUV_lesion_/SUV_normal uptake_) (mean ± standard deviation) for the clinical data

Method	SUVR	Area				
		Skull	Sternum	Ribs	Spine	Pelvis
OSEM	Max	8.6 ± 0.9	4.1 ± 1.0	7.6 ± 1.3	4.3 ± 1.9	3.8 ± 1.5
	Mean	8.8 ± 0.5	4.3 ± 1.0	7.8 ± 1.1	4.6 ± 2.2	3.9 ± 1.6
RDP	Max	9.7 ± 2.1	4.8 ± 1.4	10.1 ± 2.4	4.7 ± 2.1	4.1 ± 1.6
	Mean	10.0 ± 1.3	5.3 ± 1.4	10.5 ± 1.9	5.0 ± 2.4	4.3 ± 1.7
AMAP-S	Max	10.0 ± 2.1	5.1 ± 1.1	10.0 ± 1.2	5.7 ± 1.9	5.2 ± 1.5
	Mean	10.4 ± 1.5	5.3 ± 1.0	10.2 ± 0.8	5.8 ± 1.9	5.2 ± 1.7
AMAP-R	Max	10.5 ± 2.2	5.3 ± 1.0	10.6 ± 2.0	6.3 ± 2.2	5.4 ± 1.5
	Mean	11.3 ± 1.6	5.5 ± 1.1	10.5 ± 0.8	6.4 ± 2.1	5.4 ± 1.6

## Discussion

We compared Bayesian reconstruction methods utilizing anatomical prior information from CT (AMAP-S and AMAP-R) to Bayesian reconstruction method without anatomical information and OSEM. Analysis of the artificial lesion data show that Bayesian reconstruction with anatomical prior information improves the accuracy of SUVs when compared to OSEM. We can assume that this is also true for clinical data. Both the artificial lesion study and clinical data agree that AMAP-R method produces the most accurate, or highest, lesion SUVs and OSEM the least accurate or lowest (Tables 2, 5, 6, and 7). Bayesian reconstruction also increases the normal bone SUV, but still SUVRs obtained with Bayesian reconstruction are significantly higher than OSEM’s. This indicates better lesion detection ability. Interestingly, RDP partly outperforms AMAP-S in the artificial lesion study when lesions are not present in the CT whereas AMAP-S produces higher SUVs and SUVRs with the clinical data. A possible explanation for this is the lesion size. Artificial lesions were small, and thus, the resolution improvement provided by RDP had a bigger effect on the SUVs than in the clinical study where also larger lesions were analyzed.

AMAP-S and AMAP-R reconstructions with matching CT lesions produce the most accurate results (Table 2) especially in terms of SUV_mean._ They preserve the correct lesion size and shape (Figs. 2 and 3). The appearance of lesions is overall quite different with AMAP-S and AMAP-R when compared to OSEM or RDP (Figs. 6 and 7). Reconstructions with anatomical prior information force bone lesions in SPECT images to follow bones and not to extend outside the bone boundaries (Fig. 7).

Reconstruction with anatomical prior is susceptible to misalignments between the anatomical and emission images to a greater extent than reconstruction without anatomical prior, and this was also observed in our study. Table 3 shows that SPECT/CT misalignment has a significantly bigger effect on lesion SUVs when AMAP-S or AMAP-R is used compared to OSEM or RDP. Therefore, careful attention must be paid to the SPECT/CT alignment when using AMAP-S and AMAP-R, more so than is typically the case when CT is used only for attenuation and scatter compensations. Fortunately, SPECT/CT misalignments in bone SPECT/CT studies are usually less than 5 mm [3], which according to Figs. 4 and 5 do not produce artifacts which can be visually detected.

Bayesian reconstruction with anatomical priors has not been widely studied in SPECT. Bruyant et al. [15] found that anatomical information, which includes lesion boundaries, improves lesion detection performance. This work was based on mathematical phantom modeling of the Ga67-isotope. The same group reported similar findings in their more recent paper [16], which included more realistic simulations with mathematical phantoms and the same isotope. Kulkarni et al. [17], on the other hand, did not observe any benefit from use of anatomical prior information in a lesion detection task. Anatomical priors used in [15–17] were slightly different than those used in the present study. Priors similar to the ones used in this study have been tested in brain PET/MRI [5, 6]. They have been shown to outperform OSEM in terms of quantitative accuracy, which was also noticed in our study.

A large variety of reconstruction methods utilizing anatomical information have been presented in the literature [5–7, 14–18]. They differ in terms of the energy function and also on how the anatomical information is incorporated into the emission data reconstruction algorithm [19]. The two Bayesian algorithms utilizing anatomical information AMAP-S and AMAP-R, used in this study, have been well studied previously. They are relatively easy to implement and use, because they are dependent only on a couple of free parameters (number of nearest neighbors, Bayesian weight, and *γ* in RDP), which are not difficult to fine-tune. These two algorithms also do not require segmentation of the anatomical image, which further simplifies their use. Despite their lower complexity, AMAP-S and AMAP-R have performed well when compared to other anatomical priors [5–7]. These evaluations were, however, based on brain PET/MRI, and the results may not be directly extrapolatable to bone SPECT/CT. Therefore, testing a wider range of anatomical priors in bone SPECT/CT would be a worthwhile topic for future research.

Bayesian reconstruction methods have been available in SPECT and PET for a long time, but they have not found their way into clinical routine until lately, due to the introduction of RDP in PET [8]. Bayesian reconstruction has been considered mainly as a noise reduction technique. Images reconstructed with Bayesian methods have been criticized as looking “patchy” [20], and post-filtered OSEM-based reconstruction has been preferred by many. Utilization of anatomical information in Bayesian reconstruction, however, allows further resolution and quantitative accuracy improvements, which would be challenging to achieve with OSEM alone. This might pave the way for wider clinical adoption of Bayesian reconstruction methods in the future.

## Conclusion

We have compared two Bayesian reconstruction methods utilizing anatomical information to OSEM and Bayesian reconstruction without anatomical prior. The Bayesian methods with anatomical prior, especially the relative difference prior-based method, outperformed OSEM and reconstruction without anatomical prior in terms of quantitative accuracy. Therefore, Bayesian reconstruction with anatomical prior could be a suitable alternative for bone SPECT/CT reconstruction.

## Data Availability

The datasets used during the current study are available from the corresponding author on reasonable request.

## Abbreviations

SPECT: Single-photon emission computed tomography
CT: Computed tomography
PET: Positron emission tomography
OSEM: Ordered subsets expectation maximization
RDP: Relative difference prior
AMAP-S: Anatomically guided smoothing prior
AMAP-R: Anatomically guided relative difference prior
SUV: Standardized uptake value
MRI: Magnetic resonance imaging
ML-EM: Maximum likelihood expectation maximization
VOI: Volume of interest
OSL: One step late
FWHM: Full width at half maximum
